# From good intentions to unexpected results — a cross-scale analysis of a fishery improvement project within the Indonesian blue swimming crab

**DOI:** 10.1007/s40152-022-00285-y

**Published:** 2022-10-06

**Authors:** Sofia Käll, Beatrice Crona, Tracy Van Holt, Tim M. Daw

**Affiliations:** 1 Global Economic Dynamics and the Biosphere, The Royal Swedish Academy of Sciences, Stockholm, Sweden; 2 Stockholm Resilience Centre, Stockholm University, Stockholm, Sweden; 3 Insight Network, LLC, New York, NY USA

**Keywords:** Institutional entrepreneurship, Fishery improvement projects, Blue swimming crab, Seafood, Sustainability, Market interventions

## Abstract

Private actors have become prominent players in the work to drive social and environmental sustainability transitions. In the fisheries sector, fishery improvement projects (FIPs) aim to address environmental challenges by leveraging the capacity of industry actors and using value chains to incentivize change. Despite globally rising FIP numbers, the incentive structures behind FIP establishment and the role of internal dynamics remain poorly understood. This paper uses institutional entrepreneurship as an analytical lens to examine the institutional change surrounding the management and trade of the Indonesian blue swimming crab and sheds light on how global market dynamics, local fishery dynamics, and value chain initiatives interact to affect the trajectory towards sustainability over time. We contribute to the institutional entrepreneurship framework by extending it with social-ecological dynamics, different actors’ ability to realize or resist change, and outcomes of institutional change. These additions can improve its explanatory power in relation to sustainability initiatives in fisheries governance and beyond. Our cross-scale historical analysis of the value chain shows not only the entrepreneurship behind the FIP’s establishment, and its institutional interventions, but also why these have been unsuccessful in improving the ecological sustainability of fishers’ and traders’ behavior. This provides valuable empirical grounding to a wider debate about industry leadership and private incentives for sustainability at large and helps disentangle under what conditions such initiatives are more (or less) likely to have intended effects.

**Supplementary Information:**

The online version contains supplementary material available at 10.1007/s40152-022-00285-y.

## Introduction

In an era of globalized markets, the governance approach to addressing environmental food system sustainability has increasingly turned to private standards and multi-stakeholder partnerships (Oosterveer and Sonnenfeld 2012). Policies and strategies to “transform the market” are commonly embraced by NGOs, the private sector, and governments, highlighting that value-chain actors are nowadays viewed as possible key drivers for sustainability transitions (Bush et al. 2015; Ponte and Cheyns 2013). Alongside this trend, recent scholarship argues that firms have the potential to be important change agents in food systems (e.g., Gordon et al. 2017; Österblom et al. 2015; Folke et al. 2019), indicating the need for research on opportunities and barriers for industry leadership to realize this role. Being a change agent requires deliberate strategies and actions. Institutional entrepreneurship (sensu Garud et al. 2007; DiMaggio 1998) is a field of inquiry focused on understanding system change, and it offers a way to grasp how and why new organizations, practices, or institutions emerge and are established over time (Bakir and Jarvis 2018), increasingly also in relation to sustainability transitions (Westley et al. 2011, 2013; Rosen and Olsson 2013). In this paper, we use it to understand how the seafood industry is addressing environmental sustainability and attempting to drive change through “fishery improvement projects” (FIPs).

The FIP model was developed by NGOs in the early 2000s as a structured way for value-chain actors to engage in management issues in fisheries they sourced from. The aim was to advance the ecological status of fisheries (Cannon et al. 2018). Historically, the term was more widely used to describe a variety of projects types but, as the FIP model expanded, major conservation organizations promoting FIPs (notably Sustainable Fisheries Partnership (SFP) and WWF) worked together under the umbrella platform called Conservation Alliance for Seafood Solutions (henceforth ‘the Alliance’) to develop a common definition in 2012 (Deighan and Jenkins 2015). Today, FIPs are defined as “a multi-stakeholder effort to address environmental challenges in a fishery. These projects utilize the power of the private sector to incentivize positive changes toward sustainability in the fishery and seek to make these changes endure through policy change” (Conservation Alliance 2019: 7).

Since the implementation of the earliest FIPs, their numbers and importance in the global seafood landscape have rapidly increased. Approximately 275 projects have been initiated globally, and today, active FIPs represent around 9% of the total global wild-caught catch (FIP-DB 2021; CEA 2020). The popularity of the model can be partly explained by the overall increase in numbers of commitments among industry actors to source from sustainable and certified fisheries (Bailey et al. 2018; Roheim et al. 2018). While the Marine Stewardship Council (MSC) certification is the most widespread certification scheme for wild-caught fisheries, its standard can be difficult for fisheries to reach, particularly for small-scale fisheries or fisheries in regions with low institutional capacity for regulations (Bush et al. 2013; Ponte 2012). FIPs are therefore implemented in many fisheries that do not meet the MSC standard but have the ambition to do so. For seafood retailers, FIPs are seen as a viable sourcing option to deliver on their sustainability commitments, and fisheries within FIPs can maintain market access while working on improvements (Deighan and Jenkins 2015).

However, with increasing market recognition, the FIP model has been critiqued for potentially lowering the bar of sustainable-labeled seafood (Deighan and Jenkins 2015; Sampson et al. 2015). The Alliance therefore developed guidelines with a set of criteria that FIPs need to meet (Conservation Alliance 2019). For example, while NGOs, researchers, and governments can be included, FIPs must have active participation from the private sector (e.g., suppliers, retailers, or fishing industry). A FIP must also start with an assessment of the fishery’s environmental performance. Based on the assessment, FIP participants then develop a workplan including FIP objectives, time-bound actions, and an associated budget. To assess if projects are making credible improvements, FIPs need to report progress in relation to the workplan and to the MSC standard. All documentations, except budgets, should be publicly available. Since 2016, over 95% of FIPs publicly share their self-reported progress every 6 months on the web platform www.fisheryprogress.org (Conservation Alliance 2019; FisheryProgress 2019).

Recent research shows the importance of internal FIP dynamics for fisheries improvement outcomes and notes that despite being extensively promoted, the incentive structures behind FIP establishment and development remain poorly understood (Crona et al. 2019; Thomas Travaille, Lindley, et al. 2019; Deighan and Jenkins 2015; Packer et al. 2020). Although studies have shown that FIPs can advance fisheries management and result in positive ecological outcomes (Crona et al. 2019; CEA 2020; Cannon et al. 2018; Thomas Travaille et al. 2019a, b), they and similar marked-based incentives, overall, have been criticized for not delivering long-lasting positive improvements (Jacquet et al. 2010; Sampson et al. 2015), being unsuitable in small-scale fishery settings, and not generating benefits across the whole value chain (Barr et al. 2019; Stoll et al. 2019; Zelasney and Ford 2020). Lack of measurable success of some FIPs has been blamed on low incentives for fishers to change behavior (Tolentino-Zondervan et al. 2016) and a disregard for the social-ecological systems dynamics in which FIPs are operating (Sampson et al. 2015). The latter relates to a much wider call among scholars who argue that better understanding of social-ecological system dynamics is needed to implement fishery management effectively and reduce the risk for unintended impacts (e.g., Basurto et al. 2017; Degnbol and McCay 2007; Kittinger et al. 2013).

This paper provides an in-depth evaluation of a large and long-running FIP — Indonesian blue swimming crab — to shed light on how global market dynamics, value chain initiatives, and local fishery dynamics interact to affect the trajectory towards sustainability. Our analysis explicitly examines evidence of ecological change over time, how these ecosystem signals are noticed and acted upon by actors in the system, and the outcomes emerging from these responses. In doing so, we aim to show the explanatory importance of the social-ecological context in which FIP development happens.

The case study represents features and challenges shared by many FIPs. It is a small-scale fishery, yet connected to a big export market, with over 70% of Indonesian blue swimming crab (*Portunus pelagicus*) (from here on referred to interchangeably as crab or BSC) currently exported to the USA (SFP 2020). The crab fishery and FIP are thus representatives of the increasing numbers of seafood value chains spanning diverse geographic contexts (FAO 2020; Ponte et al. 2019) and exemplify the common situation where lead firms in the Global North work to implement change in small-scale fisheries in the Global South. As such, it captures the complexity that results when global trade intersects with small-scale fisheries where institutions are often weak, data availability low, and seafood certification standards hard to meet (Drury O’Neill et al. 2019; Crona et al. 2015a, b; Ponte and Sturgeon 2013).

**Fig. 1:**
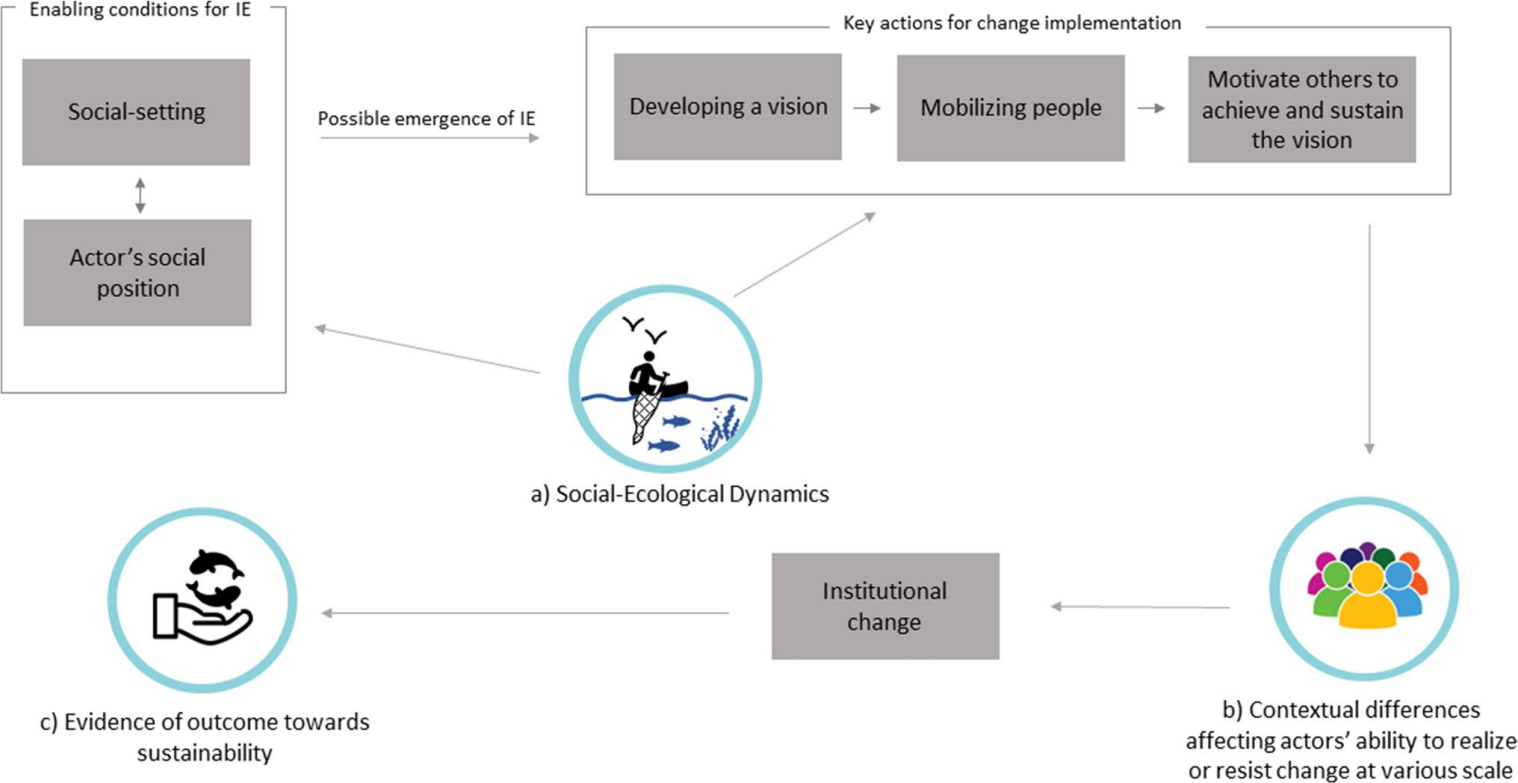
Conceptual framework for understanding the process of institutional entrepreneurship (IE) for promotion of sustainability in seafood value chains. Adapted from Battilana et al. (2009). Our expansion of the original model is represented by blue circles (**a**–**c**)

The paper is structured as follows. We first develop a conceptual framework that guides our analysis (Fig. 1) by building on the concept of institutional entrepreneurship and connecting it to social-ecological systems thinking (Folke 2016). Next, we present our case study and analytical approach which includes multiple scales across the value chain: from the USA, where the demand originates, to national Indonesian FIP system where many governance decisions are taken, and finally to the level of a local Indonesian fishing village where FIP strategies collide with the reality of social-ecological complexity. Results are presented in two parts. First, we provide a 20-year historical account of the crab fishery and FIP development, illustrating the collaborative journey of involved seafood companies and their accomplishments over time. We find that despite these improvements, strategies have not yet altered behavior by local fishers and, in fact, many fishers recently shifted fishing gear (from trap fishing to trawl fishing), thus undermining FIP measures to improve the ecological status of the fishery. The second part of the results section zooms in on the level of a fishing village, to explore in-depth why this gear change happened and likely explanatory reasons for why FIP strategies have not achieved lasting institutional change at a local level. In our discussion, we reflect on how the institutional entrepreneurship framework can shed light on enabling conditions for FIP establishment, actions for change implementation, and barriers for institutional entrepreneurship and change towards seafood sustainability. While not the primary aim of the paper, we contribute to the institutional entrepreneurship framework by extending it with social-ecological dynamics, different actors’ ability to realize or resist change, and outcomes of institutional change. Thus, this paper also increases the applicability of institutional entrepreneurship as a theoretical framework to understand processes geared towards improvements in sustainability, in fisheries and beyond.

## Theoretical background: institutional entrepreneurship and sustainable seafood value chains

FIPs intend to change institutions, including both formal and informal rules and norms (sensu North 1990) that guide behavior or organizational structures (Conservation Alliance 2019). Earlier work has evaluated the development and formation of FIPs (Thomas Travaille, Crowder, et al. 2019; Deighan and Jenkins 2015) but has not paid attention to the role of change agents in these processes, or why specific improvements were chosen over others. To understand how such deliberate shifts in institutions and practices occur, scholars have highlighted the crucial role of institutional entrepreneurs (Hardy and Maguire 2018; Fligstein 1997). We draw on this scholarship to theorize the role of change agents in FIPs. Institutional entrepreneurs are commonly defined as “actors who have an interest in particular institutional arrangements and who leverage resources to create new institutions or transform existing ones” (Maguire, Hardy, and Lawrence 2004: 657).

Sustainability scholars have increasingly applied institutional entrepreneurship to understand transformations towards sustainability (e.g., Westley et al. 2011, 2013; Rosen and Olsson 2013; Stoll 2017; Gelcich et al. 2010). However, its potential for understanding private governance and industry leadership remains largely unexplored, even though these are now important aspects of today’s global sustainability movement. Based on a review of the management literature, Battilana et al. (2009) outline a model of the institutional entrepreneurship processes that accounts for both the conditions under which it emerges, and the activities institutional entrepreneurs deploy to create change. Below we outline this model and explain how we extend it to improve its applicability for studying sustainability initiatives and processes (Fig. 1).

### Enabling conditions for institutional entrepreneurship

According to Battilana et al. (2009), the conditions enabling institutional entrepreneurship include the (1) social settings in which actors interact over a common practice, institution, issue, or goal, as well as (2) an actor’s social position within that space (Fig. 1). First, within the social setting, Battilana et al. (2009) focus on three characteristics: the degree of institutionalization i.e., the extent to which institutions govern social behavior; the diversity of institutional arrangements (different organizational forms) that institutional entrepreneurs can use to gain access to and generate new influence (Dorado 2005; Westley et al. 2013); and crises or shocks that interrupt consensus, open pathways, and provide conditions for new ideas to take shape (Dorado 2005, 2013; Greenwood et al. 2002).

The second key enabling condition is an actor’s social position. Social position influences how actors understand the social context, as well as their capacity to leverage resources and networks for change (Battilana 2006). It affects the likelihood of actors engaging in institutional entrepreneurship, as well as the ease with which change can be implemented (Dorado 2005; Maguire, Hardy, and Lawrence 2004; Battilana et al. 2009). The social position that actors hold in relation to others also shapes the validity of the strategies deployed, as perceived by others.

### Key actions for change implementation

The actions institutional entrepreneurs undertake to implement change can be broadly summarized as “developing a vision,” “mobilizing people,” and “motivating others to achieve and sustain the vision” (Battilana et al. 2009: 78) (Fig. 1). Developing a vision includes activities that make sense of the proposed change, which involves the definition of the problem, the formulation of solutions and strategies, and how to present them to interest others. Mobilizing people entails gathering crucial support from other actors, amassing followers, and creating alliances and cooperation (Battilana et al. 2009; Neil Fligstein 2001; Lawrence et al. 2002). Mobilizing people generally also encompasses drawing on personal resources and social position to gain access to financial or social resources such as access to information and political support (Battilana et al. 2009). Finally, motivating others to achieve and sustain the vision requires implementing the vision that leads to changed practices and effectively institutionalizing the change (Battilana et al. 2009). This last phase has been the least studied in the institutional entrepreneurship literature and in relation to FIPs.

### Institutional entrepreneurship in the context of sustainable seafood — an expansion of existing theory

We expand Battilana et al.’s (2009) model by incorporating three additional dimensions to understand institutional entrepreneurship in the context of sustainability initiatives: (i) social-ecological dynamics, (ii) contextual differences affecting actors’ ability to realize or resist change at various scales, and (iii) analysis of the outcomes of institutional change (Fig. 1a–c).

While it is clear that both the social setting and position of actors affect their capacity to steer change, the interplay between social and ecological components of the system to be changed has not featured strongly in the institutional entrepreneurship literature (Hardy and Maguire 2018). We propose that an actor’s connections to various natural resources play a role for ecological knowledge (ecological literacy) by affecting incentives and willingness to act (Crona and Bodin 2010, 2006; Stoll 2017). For institutional entrepreneurs pursuing sustainability, social-ecological knowledge can also minimize the risk that new institutions or practices unintendedly become destructive for ecosystems or social wellbeing (Rosen and Olsson 2013). Seafood value-chain actors are ostensibly well-positioned to receive signals from the ecosystem (Crona et al. 2010; Österblom et al. 2015), but this may be “masked, diluted and drowned out” by international seafood trade (Crona et al. 2015a, b). We, therefore, extend the model to include how institutional entrepreneurs perceive ecosystem signals and how this influences their ability to create problem awareness, implement actions, and shape actors’ behavior (Fig. 1a).

Our second extension of the model accounts for how social context influences actors’ ability to realize or resist change at various scales. FIPs — along with multiple other private governance initiatives — aim to incentivize change throughout the value chain. Studies highlight the limitation of top-down approaches applied in many FIPs, because the approach “effectively ‘pushes down’ the responsibility, with more powerful stakeholders in the value chain passing sustainability responsibilities down to those who are less powerful” (Zelasney and Ford 2020: 161). To capture such dynamics, we introduce an analysis of how institutional change created by the FIP plays out among other actors in the value chain, in our case spanning from importers and exporters to local fishers and traders (Fig. 1b). Our proposition is that local context and views from extracting actors may explain resistance to the vision and practices of institutional entrepreneurs higher up in the FIP organizational structure. Understanding these potential barriers to lasting change is important, but has been underexplored in the literature (Hardy and Maguire 2018).

Finally, recent literature argues that earlier institutional entrepreneurship work failed to problematize new institutional arrangements that have emerged and uncritically assume that they improve the earlier situation (Hardy and Maguire 2018; Khan et al. 2007). We consequently emphasize the importance of examining the ultimate effects of institutional change and analyze whether FIP-related initiatives further a more sustainable fishery, represented as the arrow between institutional change and evidence of outcome towards sustainability in Fig. 1. “Sustainability” in our framework refers to seafood production that meets the needs of the present without compromising the ability of future generations to meet their own needs (WCED 1987; Clark and Harley 2020). We restrict our focus to the environmental pillar of sustainability, and, more specifically, we analyze sustainability outcomes as a function of harvesters’ interaction with the ecosystem.

## Methods

### Research approach

Our research approach is inspired by multi-sited ethnography because it allows us to “follow something through different geographic or social fields in order to grasp a given event, topic, or process” (Nielsen et al. 2019:308). By doing so, we triangulate perspectives on the fishery history and FIP actions and outcomes from different actors in the value chain (Marcus 1995; Nielsen et al. 2019). Our data collection and analysis spans multiple scales, including the context where demand originates (the USA), the fishery and FIP system at the national Indonesian level, and place-based research at the local level, using the fishing village of Betahwalang as a case-within-the case. This allows us to provide a general analysis of the fishery and FIP, as well as a situated analysis of how the FIP intersects with local dynamics in a place of extraction.

### The case study

#### Indonesian blue swimming crab fishery and the FIP

The BSC fishery is the third most economically valued seafood industry in Indonesia, and the country is the largest producer in the world providing around 25% of the global BSC supply (CEA 2018). Crab exports began around 1994 and were valued at USD 321 million in 2016, with approximately 52,000 tons landed annually (SFP 2020). Over 70% of crab catches are exported to the USA, via a group of around 30 importing companies. It is a small-scale fishery that employs around 90,000 fishers and 185,000 pickers working in over 550 local processing plants nationally (APRI 2022; CEA 2018). While fishing is most intensively pursued in the Java region, extraction occurs throughout the Indonesian archipelago, especially in Sulawesi and Kalimantan (SFP 2020). Henceforth, when referring to the BSC fishery, we refer to the market and the markets actors that emerged after exports began.

The FIP was established in 2009 by an Indonesian Crab Processing Association (IPA), a US-Crab Processing Association (USPA), and an international environmental NGO (IENGO). The FIP is now led by the IPA and implemented at a national scale throughout Indonesia. The FIP started because of signs of overfishing, with the long-term goal of reversing this trend. Table 1 summarizes the objectives and key FIP outputs reported. The FIP’s work has mainly focused on collecting ecological data, working with government authorities to establish management measures, changing fishing and processing practices through industry schemes, and creating awareness (for an overview of FIP activities, outputs, and workplans, see Appendix 1).

**Table 1:** Summary of information of the Indonesian blue swimming crab FIP (FisheryProgress 2022; Aravind 2020; APRI 2018)

Year started	2009
Initiating organizations	Indonesian Crab Processing Association (IPA)
	U.S. Crab Processing Association (USPA)
	International environmental NGO (IENGO)
FIP lead	2013–current: IPA with full support from USPA
	2009–2013: IENGO with full support from USPA and IPA
FIP participants	IPA
	USPA
	Collaboration with several other organizations (e.g., IENGO, universities, Indonesian governmental agencies, UNDP)
FIP performance^a^	Comprehensive FIP^a^
	Reports at fisheryprogress.org since 2016
	FIP stage 5: improvement on the water
	Progress rating: A
Funding	Receives funding from the USPA, IPA and other external funding
Objectives (to be met by the end of 2022)	1. Implement FIP workplan and transition Indonesia Blue Swimming Crab Fisheries to MSC full assessment
	2. Change practices (no-take of small crabs/juveniles, no take of egg-bearing females), increase the stocks, develop policies that protect and sustain crabs (including protection of nursery ground, spawning area)
	3. Develop a community-based management plan for Indonesian blue swimming crab that includes community resources management that protects nursery ground, communication and awareness, produce and established control document and traceability system
Summary of key reported outputs until the end of 2019	• Stock assessments in the Java Sea
	• Systematic collection of ecological data with deployed enumerators
	• National Management polices
	*- Blue Swimming Crab Fishery Management Plan that prescribes the Road Map for sustainable management. (NOMOR 70/KEPMEN-KP/2016)*
	*- Minimum landing size and prohibition on landing/processing of egg-bearing female (56/PERMEN-KP/2015)*
	*- Fishing gear ban on coastal and seine-trawls (No.2/PERMEN-KP/2015)*
	• Several Co-management regulations on regional and local scale (e.*g., Betahwalang village BSC management decree (NOMOR:06/2013 PerDes No.02/2019); Demak mayor decree on BSC fishery management in Demak Regency (NOMOR 523/0166/ 2014); Fishery Management Committee in Central Java.)*
	• Control document in place, an industry self-regulation traceability scheme to increase compliance to government polices
	• Awareness campaigns towards fishers about crab ecology and sustainable fishing practices

^a^The Alliance categorizes FIPs into two types, either basic or comprehensive. Basic FIPs focus on a subset of environmental issues to improve upon, whereas comprehensive FIPs address all environmental matters covered under the MSC Fisheries Standard and often have an end goal of achieving certification. Comprehensive FIPs must also have an independent audit of their progress against the MSC standard every third year (Conservation Alliance 2019). The FIP process is divided into a stepwise progress with five different stages: (1) FIP development, (2) FIP launch, (3) FIP implementation, (4) improvements in fishing practices or fishery management, (5) improvements on the water. (For more information see Conservation Alliance 2019). Progress rating A refers to a comprehensive FIP that have achieved a stage 4 or 5 result within the past 12 months

#### The fishing village — Betahwalang

**Fig. 2:**
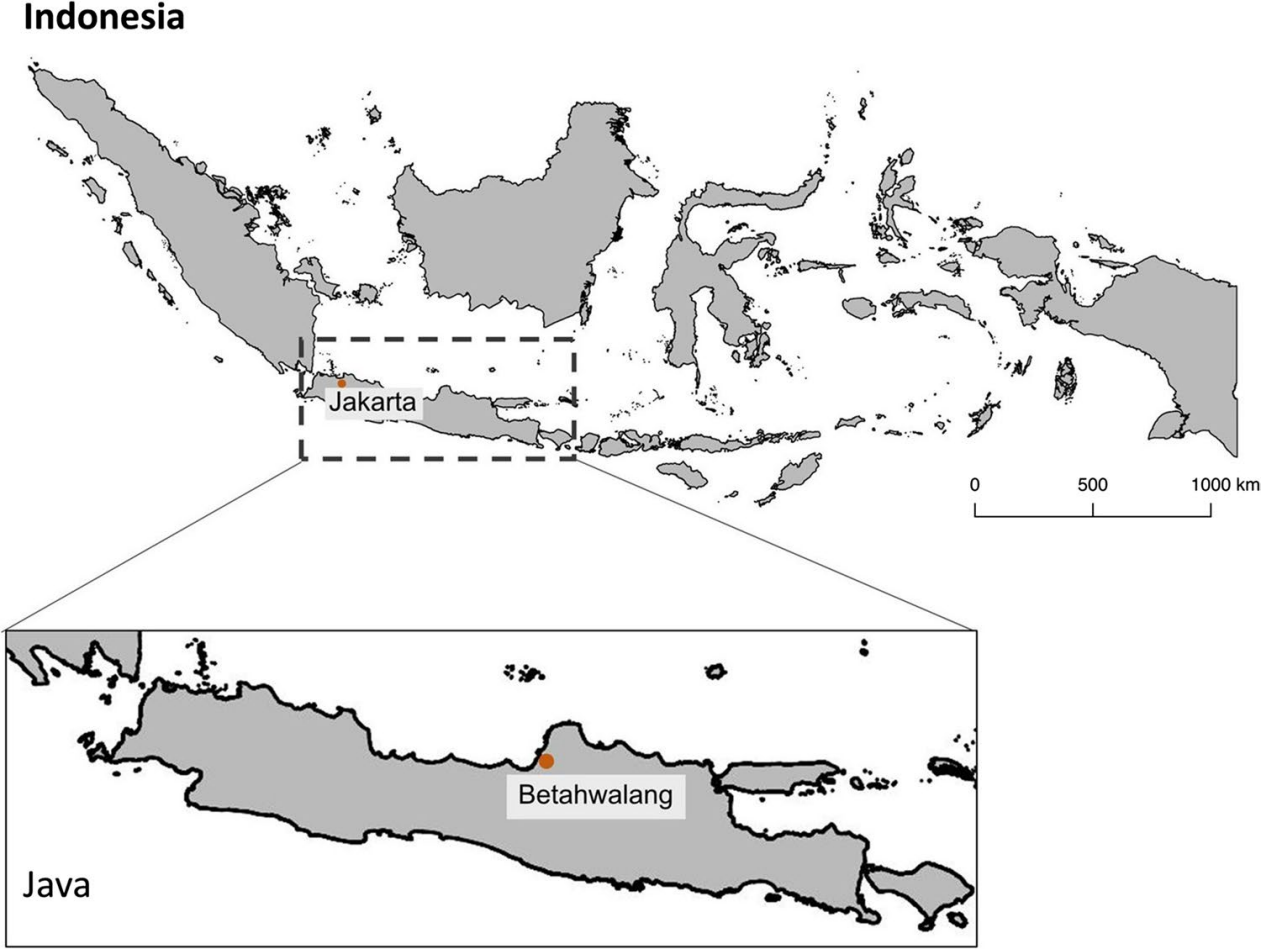
Map of Indonesia showing the case study site Betahwalang on the island of Java

To understand how FIP strategies intersect with local social-ecological dynamics, we focus on a fishing village known for its BSC fishery. Betahwalang is located in Demak Regency in the Central Java Province (Fig. 2). The village has around 5700 inhabitants, of which approximately 24% are fishers. Village fishers shifted to BSC fishing when the export fishery was first introduced to Indonesia in the mid-90s, and crab fishing is now a key livelihood for many villagers (Ghofar et al 2018). The village produces an average of 253 tons a year of BSC (Prabawani 2018).

FIP actors have been particularly engaged in Betahwalang since the start of the project. For example, emerging from the engagement of the FIP and its collaborative university partner, a village regulation for BSC management came into place in 2013 to establish a protected area for crab habitat and was extended in 2019 to include spawning areas (Redjeki et al. 2020). The village was highlighted by the Indonesian government for its work towards sustainability and has become a national icon of a BSC village.

#### Data collection

We use qualitative methods based on in-depth interviews conducted by the lead author. First, we interviewed key informants representing industry and NGOs in both Indonesia and the USA, chosen for their prominent leadership roles in the FIP over time. These interviews were carried out in multiple locations in the USA, as well as online. Second, a fieldwork campaign in Betahwalang in Indonesia included interviews with fishers, traders, processors, and NGO actors. Interviews centered on open-ended questions adapted to the specific type of actors, but all focused on three main themes: (1) experiences of FIP processes; (2) trading, fishing practices, and value chain relationships; and (3) perceptions of problems and solutions for sustainability within the fishery. We conducted several follow-up interviews with key actors for a deeper understanding of the case and individuals’ perceptions, strategies, and motivations. In total, 61 interviews were conducted with 45 people between January 2018 and August 2019. Interviews lasted from 20 min to 3 h and were done in English as well as in Bahasa Indonesia and Javanese with the help of an interpreter. Interviews were recorded based on consent, translated, and transcribed. If an interview was not able to be recorded, extensive notes were taken.

Participant observations were carried out in Betahwalang, at landing sites and markets, and during fishing trips to observe fishing and trade practices. Fishing group meetings were also attended to add perspective on issues that were discussed among fishers in the community. In addition, one focus group interview was held with fishers and traders, early in the fieldwork to flag topics of importance that needed to be captured in the interview guide. Moreover, we conducted participant observation in the USA at a large seafood trade event with many crab industry actors present, as well as at a famous Maryland Crab restaurant. Finally, we collected secondary data, including publicly available FIP reports and information. These documents were analyzed to understand actions reported by the FIP over time and were triangulated with findings from other data collection methods (see Appendix 2 for a summary of data collection methods). The study was reviewed for research ethics by the Stockholm Resilience Centre Research Ethics Sub-Committee.

#### Data analysis

**Fig. 3:**
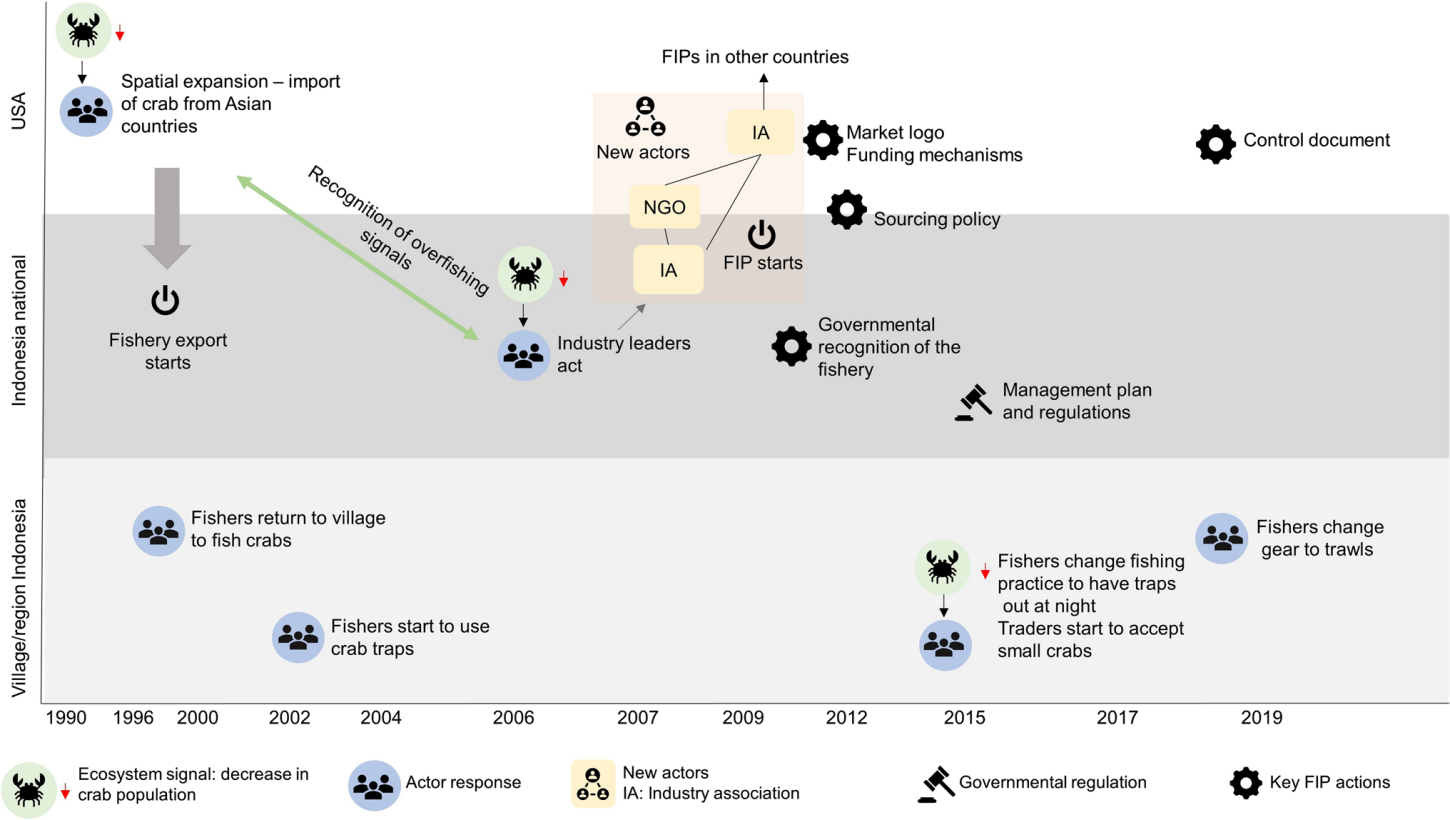
The development and key events of the fishery and FIP on three different geographical levels: USA, national, and village/regional level in Indonesia

The analysis is divided into two parts. The first is an in-depth historical account to understand how and why industry actors got involved, how the FIP emerged and was implemented (Fig. 3). We coded all interview transcripts, notes, and secondary data using MaxQDA. The first coding round applied an inductive approach to explore the data by structuring it into a broad timeline of major trends and events in the fishery and FIP across the different scales (Saldaña 2013). A second coding round followed the conceptual framework (Saldaña 2013; Fig. 1), to understand causal connections and outcomes (Fig. 3). We specifically looked for evidence of institutional entrepreneurship among the key actors in the FIP, drawing on Maguire et al.’s (2004: 657) definition: “actors who have an interest in particular institutional arrangements and who leverage resources to create new institutions or transform existing ones.”

**Fig. 4:**
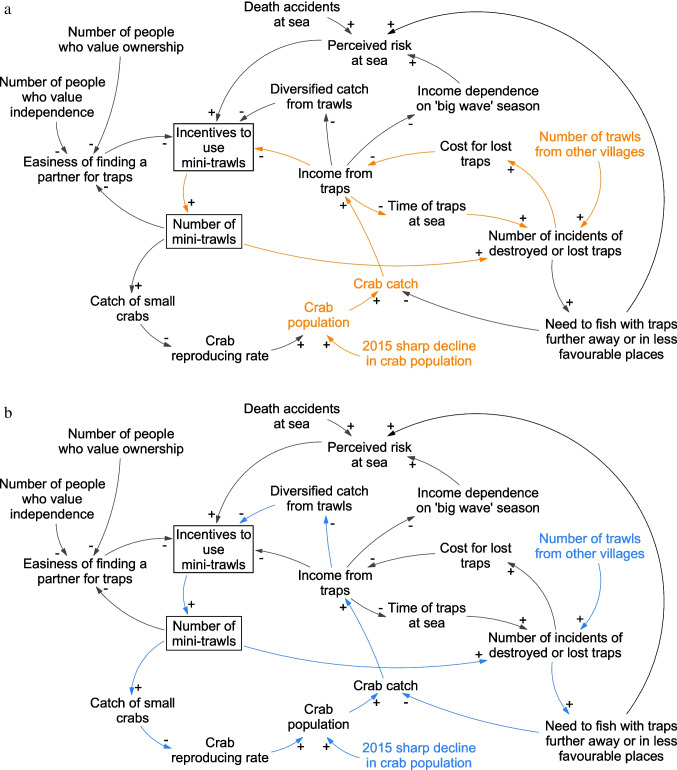

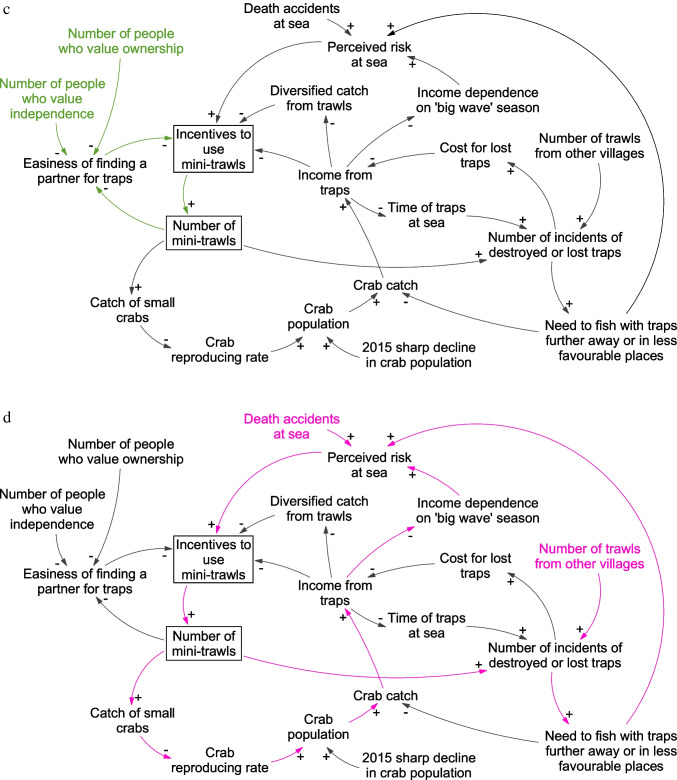
Causal loop diagram showing four dynamics for why fishers change from fishing with traps to trawls. (**a**) Lost traps (orange arrows); (**b**) diversified catch (blue arrows); (**c**) finding a fishing partner (green arrows); (**d**) risk at sea (pink arrows). Variables in the same colors as the arrows indicate that they are the main drivers

In the second part of the analysis, we synthesized our data on local-level social-ecological interactions and reasons for changes in fishing practices, using causal loop diagrams (Fig. 4). Causal loop methodology helps to structure analysis and examine how multiple causal relations may interact (de Pinho 2015). It is a first step towards unpacking the complex causality that potentially explains the development of the fishery, including feedback loops that affect the potential for change within the system (Downing et al. 2014). Having developed the causal loop diagram from our coded interviews and observations, we presented it to NGO FIP practitioners working within our case study to validate the findings of the model.

## Results

### Historical account

#### *“*Maryland is crazy about crab*”* — a local resource becomes a global commodity

The globalized market and trade of the Indonesian blue swimming crab fishery started on the east coast of the USA, with a restaurant pioneer (“Brad”) from Maryland. Understanding Brad’s story and the Maryland context is key to understanding the development of the Indonesian crab fishery. Crabs are part of the local Maryland identity, and the region is famous for its iconic Maryland-style crab (Paolissom 2007). Brad was part of a fishing family and often fished crabs with his grandfather “at a very young age I learned about the crab industry, I was just fascinated with it, I loved it (…). You know I got saltwater in my veins, I think” (interviewee #3). The family later opened a small seafood restaurant, which grew into a restaurant chain, and eventually also a seafood processing company. Directly after leaving school, Brad began work in the family business.

In the late 1980s and early 1990s, the crab population in Chesapeake Bay declined from overfishing (Sharov et al. 2003); this made it hard for Brad to source crab. “Our restaurants grew, and I really had a hard time getting enough crab meat to satisfy the customers; because Maryland is crazy about crab.” But the crab population was threatened and with it a way of life. Brad notes; “I had no idea that in my life time I would see such a tremendous decline of a natural resource, you know which put a lot of stress on those communities (…). So, I was really, really concerned about it.”

Around this time, the import market of shrimp from Asia had started in the USA. By chance, Brad read an article about the emergent shrimp industry in Philippines and, in a photo, he spotted a bucket of crabs in the background. “They have crabs in Asia!” he concluded, and this catalyzed a journey that led to the development of the Asian export industry of crabs to the USA. Brad travelled to the Philippines to explore the possibilities for the fishery. The shrimp industry provided an existing export trade structure and political interest in seafood exports. He had meetings with the government who were very surprised he was not there for the shrimp, but for crab.

##### The value chain extends 

Brad established a Filipino company in 1990 and started to build crab processing factories. He also initiated an informal sourcing policy, as a conservation mechanism. This included the idea of having minimum size of crabs and no-take of berried females. Brad stated: “Because if I was going to do this, I didn’t want to have a situation like we had in the Chesapeake Bay where eventually the resource is overfished.” However, as the fishery grew, there was a lack of compliance with the conservation mechanisms by competing companies.

After the start of the crab export business in the Philippines, Brad expanded to Indonesia and later to other Asian countries, for example, Vietnam and Sri Lanka. The Indonesian exporting crab fishery started around 1994, and crabs were sold as picked and pasteurized products. For the first 3 years, Brad’s company was the only company exporting from Indonesia, until a former employee started a new company. This was then followed by others, and around 10 years after, there were 28 listed Indonesian crab exporting companies, with 10 main companies contributing to more than 80% of the total export (SFP 2009).

At the start of the fishery, companies bought directly from fishers, but over time, a long value chain had/was built up. From around 1999, local independent processing plants called “miniplants” were established, which sourced crabs from fishers for boiling and picking. These were developed because Brad had noticed that crab quality declined when crabs were transported fresh for long distances. Miniplants received financial resources and equipment from exporters as well as training on the specific practice of Maryland crab traditions. Some years after the development of miniplants, crab traders called “collectors” emerged in the value chain. These collectors established landing sites and started buying crabs from fishers and boiling them before delivery to miniplants. As the crab industry grew, so did the number of miniplants. Today, there are over 550 miniplants scattered around the country that operate independently from the exporting companies (APRI 2022).

##### *“*Betahwalang Desa Pengelolaan Rajungan” — a crab village is born 

Blue swimming crab is not consumed in Indonesia to any large extent. Thus, before the start of the BSC exporting fishery, fishers did not sell or target crab in Batawhalang. In fact, many fishers from Betahwalang were working in other parts of Indonesia. With the development of crab fishing, many fishers came back to Betahwalang, giving a new life to the village. This is celebrated by a welcome arch at the entrance to the village, with the text “Betahwalang Desa Pengeloloaan Rajungan — Betahwalang the blue swimming crab village.”

Crabs were initially fished with special gillnets (jaring) for crab fishing, handed out from the exporting companies to fishers to encourage them to catch crabs. But when collectors became common, they began handing out collapsible crab traps (bubu) which, over time, became the most common fishing gear in Betahwalang. In addition, some fishers started to use so-called minitrawls (arad). Although crabs are the main target species, particularly for trap fishers, fishers using trawls and gillnets catch and sell other species, especially shrimp for the trawl fishers, but also a variety of fish species depending on the season.

Betahwalang is not directly located in the open water where fishers fish, but next to a river, surrounded by mangroves, leading out to the ocean. The boat ride takes about 30 min from the village to the start of the fishing grounds. To approach fishers as early as possible from their fishing trips, collectors started to build bamboo stands in the river mouth, where they buy and steam the crabs. This system is still in place today. Around 1998, there were two collectors in Betawhalang, but numbers have gradually increased, and in 2019, 14 traders were operating. Three of them have large operations, buying from around 100 boats, while the rest are smaller buying mostly from families and friends. Most fishers only sell to one collector, whom they are often tied to through debt. The price of crab is always set by the collectors, who in turn state that the price is set by the miniplants, which are dependent on the larger exporting companies. Most collectors only sell to one miniplant. There is one small miniplant in the village owned by a local collector, but the bulk of crabs from Betahwalang are sold to miniplants in a lager city, Rembang.

#### “A marriage made in heaven” — the establishment of a FIP

After a decade of importing crab from Indonesia, Brad recognized signs of overfishing. “Crabs [were] getting smaller which is a sign that the resource is under stress.” Brad also talked to fishers who indicated they got a lower catch and had to work longer hours. With a similar experience from the Chesapeake Bay, he did not want the resource to collapse, feeling a sense of responsibility for the resource: “I said to myself, I cannot come over here and start this business and then you know in 20 years there is no crab left.(..) I have to do something about it.”

The first step Brad took was to gather exporter companies in Indonesia to form an industry association, hereon referred to as the Indonesian Processing Association (IPA) in 2007. Brad’s status in the industry helped to get people on board in the association, as many of them had been trained by him. The first seven companies that agreed to join represented the majority of the crab produced. Since then, interest has grown, and in 2019, there were 17 member companies together representing 85% of the crab volume produced. To set up an industry processing association, a law firm and a facilitator were hired to ensure that the meetings were not about price-fixing but about sustainability. The strategy was to work with a third-party NGO to make the work towards sustainability credible, Brad noted: “Not many people trust the seafood industry in regards to their efforts on sustainability.” The IPA started a close collaboration with a business-oriented international environmental NGO (IENGO) which had been promoting fishery improvement projects. The sustainability officer in Brad’s company thought that the FIP model sounded “like a marriage made in heaven” (interviewee #1), and they decided to start one.

After the formation of the IPA in Indonesia, Brad and his team decided to organize American companies in a similar way to fund FIP implementations. They called a meeting with the US-based importing companies and successfully proposed the formation of an industry association for crab sustainability, henceforth referred to as the U.S. Crab Processing Association (USPA). Initially located under Brad’s company, it was later transitioned to be under a US national trade group representing the seafood industry. One challenge for the USPA was to create its own identity and legitimacy and to not be seen as only the product of one company and one man.

Interviews and archives indicate two central reasons why companies joined the initiative: firstly, the visionary leadership by Brad, and secondly, the understanding and memory of the collapse of the crab population in the USA. As explained by a USPA member: “For those companies to recognize (…) we used to have thriving robust crab population right here right in the Chesapeake Bay, and because we mismanaged it, we don’t anymore. So, I think that, sort of having hindsight is really important” (interviewee #12). The USPA continues to work to get buyers to recognize membership of the council in their purchasing policies. From the beginning, there were seven companies; now, there are 33, representing 90% of the industry importing BSC to the USA suggesting that the strategy of market recognition has been successful. Different levels of engagement in USPA have been under discussion and have been addressed by rules for membership and a sanctioning system if companies are inactive.

A long-term funding scheme was in the minds of the pioneers of the council from the start. They implemented a tax of $0.02 per pound of imported crab meat to the USA by the member companies. This was later supplemented by funding from donor organizations, for example, the World Bank, who was impressed by the industry funding scheme. The idea behind the USPA funding system was to ease the FIP’s economic burden of the Indonesian companies in IPA, who would lead the project implementation. To create a market incentive to join the council, a logo was established that members could use on their products to show that they were “committed to sustainability.” After the establishment of the USPA and IPA, the FIP was ready to be launched.

#### “Save our crabs” — implementation and improvements

To start, the IPA and the IENGO held a big workshop in 2009 titled “Save our crabs,” with the government and universities to raise awareness of crab as an exporting commodity and why it is important to manage the fishery. At this time, there were no regulations or data collection in place for BSC. For the first 4 years, the IENGO was the lead organization of the FIP until 2013, when IPA fully took over. In this FIP, the lead organization’s role was to make workplans and specify coming actions with a proposed budget. The workplan and budget are then approved by the USPA who funds the majority of the budget for the project. In addition, the IPA also has some internal funding from member companies for FIP implementation. Below, we outline some of the key actions highlighting how the FIP’s strategy has evolved.

One of the first actions was to conduct a MSC pre-assessment which discovered that the health of the crab populations was worse than many industry actors had thought. In 2010, the IPA hired a director and the FIP focused on working with the government to recognize BSC as an important species and work towards stock assessments. The director at that time notes “I think the first four years from 2010 until 2014, the greatest achievement was that the government recognized blue swimming crab as one of the important species. The first times we met the government they didn’t even know what is blue swimming crab” (interviewee #7).

Shortly after, in 2011, the USPA declared a sourcing policy on minimum legal crab size (8 cm) and restrictions on buying berried females. The policies were then communicated among value chain actors, and the FIP conducted outreach campaigns to fishers. The theory of change relied on a belief that the fishery is “unique” and conservation measures were “self-incentivized” (interviewee #12) because larger crabs were more profitable for industry actors — as well as better practice for the ecosystem. In addition to the sourcing policy, the FIP strongly promoted the use of crab traps to fishers, with the argument that the gear is the most selective and “environmentally friendly” gear (interviewee #18) as it is less destructive to the ecosystem. Also, catch from traps often includes fewer smaller crabs, in general, and higher quality meat for processing compared to catch from trawls and gillnets. Moreover, the FIP worked with stock enhancement strategies, developed crab hatcheries, and released crab juveniles into the ocean.

The FIP worked intensely to lobby the government to regulate the fishery, and in 2012, a discussion of a BSC management plan started. They even provided their own data on the crab stock to convince the government of the urgency of managing the fishery. In 2015, the government announced regulations for BSC, imposed by the Indonesian Ministry of Marine Affair (MMAF), and included the following: a minimum legal size of 10 cm; prohibition on landing/processing of egg-bearing females; and prohibition on trawl systems (MMAF 2015).^1^ In 2016, a full national management plan of the BSC was established which functions as a road map and guidance for government at regional and local levels. The new regulations and the management plan were perceived by interviewees as a great success of the FIP. An IENGO representative noted that the regulations were “a blessing” (interviewee #11), although they could not be sure that it was the work by the FIP that was the main reason behind the government decision.

Despite the apparent success with governmental regulations in place, interviewees from the USPA, the IPA, and IENGO noted that compliance with the sourcing policy, now turned into regulations, remained low, together with a lack of governmental enforcement. This is also echoed in several assessments of the fishery (USITC 2021; SFP 2020; CEA 2018; Ghofar et al. 2018). Based on success stories in other fisheries, the IENGO then recommended the USPA and IPA to develop a so-called control document, an industry self-regulation tool with compliance audits along the value chain to eliminate illegally caught crab (see Van Holt and Weisman 2016). The control document is a contract between buyers and suppliers that should create commercial penalties, for example, loss of business, if suppliers do not follow governmental fisheries regulations.

In this case, the control document starts when companies within the USPA ask their supplier for a letter of warranty.^2^ The control document is a voluntary tool and is implemented by each of the member companies. Together, the IAP, the USPA, and the IENGO developed and designed the scheme. They implemented several trial phases from 2016 to 2018 which included feedback from actors in the value chain and companies within the processing associations. The IPA also held educational campaigns about the scheme. When it was fully rolled out among all companies in 2018, a steering committee was set up with several Indonesian governmental organizations and the initiating organizations to guide the sanctioning system of the control document, to be put in place if industry actors fail the audits (Aravind 2020). The first audits were held at the miniplant segment in 2019.

##### When the rubber hits the road — local dynamics intersect with FIP governance

When it comes to the local level in Betahwalang, the FIP initiated a management program based on co-management principles in 2013. It resulted in a local fisheries management body focusing on crab as well as a marine protected area under village legislation (Ghofar et al. 2018). In conjunction with this, the FIP worked with several awareness campaigns, including crab ecology and status, promoting the more selective fishing gear crab traps, and discouraging the use of trawls, encouraging fishers not to catch berried females and undersized crabs, as well as giving out information about procurement specifications and governmental regulations. Respondents representing the IENGO believed that all fishers and collectors in Betahwalang knew about the governmental regulations and were aware of the challenges with overfishing. Fishers also noted that they agreed with the regulations and thought they were needed.

Yet, in spite of the awareness and generally positive attitude towards the regulations and the implementation of the control document, interviewees stated that regulations were seldom followed and enforced at a local level. In particular, many fishers recently have shifted from fishing with traps to trawls, a development in the “opposite direction” of the work of the FIP which has been discouraging the gear. The use of minitrawls in the BSC fishery has been identified as a threat to the sustainability of the BSC stock since the gear is unselective, catching all sizes of BSCs, including small-sized immature crabs. Along with the catch of non-target species and damage to the seafloor, the minitrawl is recognized as an environmental destructive gear (Ghofar et al. 2018; Sara et al. 2020). While the control document has only been fully in place for a year, and it may be too early to determine its effects, our data on fishing and trade practices indicate limited effect on the ground in Betahwalang. Thus, despite intensive work by actors at multiple levels, and across diverse geographies, the social-ecological system underpinning the global crab value chain is still not on a sustainable trajectory. Why is that? The next section analyzes the interactions between local social-ecological dynamics that are not directly addressed by the FIP and related institutions, yet fundamentally impact their effectiveness.

#### A system on a trajectory towards a “small crab state”?

While the 2006 national Indonesian decline in crab seems to have triggered the responses that ultimately led to the FIP establishment, respondents in Betahwalang did not report seeing crab catch decline until around 2009, with a sharp drop in 2015 (Fig. 3). This ecological signal induced a change in fishing and trading practices in the village. Fishers, using crab traps (i.e., the majority of fishers), began increasing their fishing effort by leaving their traps in for longer, often overnight. At the same time, collectors started accepting smaller crabs because of the decline in total catch and the pressure from their buyer, the miniplants, to deliver a certain volume. Fishers reported that before the decline, they felt they caught “enough” larger crabs and could release smaller crabs at sea, a practice that reportedly became less common around 2015. An earlier study in Betahwalang supports this narrative and concludes that as of 2013, fishers did not land, and traders did not buy, smaller crabs (Bokkes 2013). Our observations and interviews with fishers and collectors indicate that in contrast, today, smaller crabs are consistently bought from the fishers, although the acceptance of undersized crabs varies with seasons. A trader explained: “Nowadays, if there aren’t any small crabs for the miniplant, the business won’t work. (..) Finally, the small ones got to be included” (interviewee #36). Furthermore, fishers maintained that they needed the income that berried females provide, as crabs with eggs are heavier and fishers are paid per kilogram.

Starting early 2018, many fishers began using trawls, mostly switching from traps. The trend has continued, and in 1.5 years, trawls went from being the least to the most used gear in Betahwalang. Whereas some fishers were quite happy with the change of gear, others felt that they had no choice even though they would prefer not to, because of personal fishing preferences and the harm trawls have on the ecosystem. Developing causal loops based on our interviews allowed us to connect fishers’ behavior with events over time and uncover plausible causal connections and reinforcing dynamics ensuing from this, such as the drivers of fishers’ gear choices (Fig. 4a–d). This shows that gear choices appear to have triggered vicious cycles that might be difficult to break and change. For example, interviewees explain that high fishing pressure led to a change in the crab population, with a higher abundance of smaller crabs and fewer larger crabs. This in turn led fishers to increase their fishing efforts and starting to land smaller crabs and possibly accelerating the decline of crab populations, as fewer crabs reach maturity. The change to trawl fishing then further accelerated the process of overfishing due to the higher catch and landings of undersized crabs, creating a reinforcing feedback loop (Fig. 4). Fishers explained that the gear change meant that the minimum size and ban on berried female make less sense to them. When fishers used traps, they could release berried and undersized crabs in the belief that the crabs will survive. However, trawled crabs are often dead when trawls are emptied and fishers noted that “if they are already dead they will only be litter” (interviewee #26). This increases the incentive to try to land all crabs. While collectors reported trying to disincentivize landings of crabs from trawls and small crabs, both by “reminding the fishers” (interviewee #27) and paying a lower price per kilogram for catches caught by trawls, declining catches seems to have made more traders inclined to buy undersized crabs to satisfy the demand, even though smaller crabs have a lower market value and are more expensive to process. Saputra (2020) notes similar trends among crab traders in another region of Java, Banyuwangi regency, in Indonesia. Changing practice among traders therefore further reinforces and incentivizes the use of trawls.

This reinforcing feedback also appears to be driven by practices from fishers not part of the BSC fishery, as well as norms and events in, or adjacent to, the village. Together, these dynamics currently lock the fishery into a “small crab state,” in turn leading to further changes in fishing practices and low compliance with regulations. This dynamic is a significant threat to the sustainability of the crab population, since the continued catch of small crabs, below first maturity, can put at risk the future of the crab populations and lead to stock collapse (Ghofar et al. 2018). Throughout interviews and observations, fishers return to four dynamics that influence their gear choices (outlined in Table 2) and which together appear to lock the fishery in a small-crab state (Fig. 4a–d) by reinforcing the use of trawls in Betahwalang.

**Table 2:** Four local social-ecological dynamics influencing gear use and potentiallyreinforcing the use of trawls in Betahwalang

Factors affecting gear choice	Contributing social-ecological dynamic
Lost traps in a crowded sea (Fig. 4a)	• At sea, traps are easily lost in currents or damaged by trawls. Leaving traps at sea overnight to compensate for lower catch rates thus increases the risk of loss • Fishers report that increased use of trawls in the area around Betahwalang, mainly by fishers targeting other species than crabs, has increased the number of traps destroyed by trawls. In addition, trawl fishers have started to fish where trap fishers usually fished, thus increasing the likelihood of trap-trawl clashes • The risk of losing traps is a stress and an economic burden for fishers, and several have shifted to trawls after losing traps, citing both the lower investment cost for trawls compared to traps and the increased struggle to fish with traps as key reasons • While fishers believed it was primarily trawl fishers from other villages that run into their traps, the higher number of trawl fishers in the village is also contributing to an increased risk of trap damage and the need to move to other fishing locations • Rising numbers of trawls in the village therefore directly increase the incentives to use trawls, creating a reinforcing feedback
Diversified catches and higher income using trawls (Fig. 4b)	• According to some fishers, trawls can generate higher returns in the context of declining crab catches. With trawls, fishing income is diversified as they catch both shrimp and crab depending on season, along with many other species such as squid and fish, securing a steady and sometimes higher income • Trap fishers have moved further from the coast to avoid clashes with trawls, but with limited success. Fishers suggested some trawlers simply do not care about running into other people’s gear. This dynamic has caused trap fishers to carefully select fishing locations based on low likelihood of trawl interference, not based on high likelihood of large catches, with further negative effects on catch rates • Rising numbers of trawl fishers are likely to reduce trap fisher incomes by putting more pressure on crab stocks and directly causing damage to traps. Thus, there is a reinforcing feedback by which more fishers switching to trawls increase the incentives for trap fishers to also switch
Difficulties finding a fishing partner and a strive for independence (Fig. 4c)	• Trap fishing requires two people on the boat, whereas trawl fishing only requires one person. It is common among trap fishers that one person owns the boat, and in the past, fishers often fished with family members • Broader changes in the norms and attitudes toward boat ownership and increased value of being independent appear to have changed this tradition. Many younger fishers want to own a boat after getting married • Boat-owning fishers, therefore, noted that it is increasingly hard to find a partner to fish with, and as a result, they cannot continue trap fishing • Fishers also noted that the lower amounts of crabs mean lower earnings which are harder to split, making trap fishing more complicated • Based on these dynamics, trap fishers complain of difficulties in finding a fishing partner, yet noted that they would rather change gear than fish with a partner outside their family network. Consequently, a self-reinforcing feedback is pushing the system away from trap fishing
Risk at sea (Fig. 4d)	• Trap fishers generally get their largest catch during the “big wave season” (Jan–Mar), but fishing during this period involves greater risk from bad weather • During this period, it is hard for minitrawls to fish meaning less trap-trawl interference at sea and larger catches for trap fishers • Declining catch rates and conflicts with trawls during other seasons have led trap fishers to become more dependent on the catch during this season • But fishing in this season is associated with higher risks. In 2018, two villagers lost their lives during the “big wave season” and this accident caused some fishers to change to trawls, for personal safety • The need for trap fishers to fish further from the shore to avoid trawls further increases the level of risk at sea, thus feeding into decisions to change gears

Together, the four dynamics outlined in Table 2 reinforce a trajectory in which trawls become the dominating gear, pushing the fishery to a small-crab state, and countering the ambitions of the FIP. Furthermore, the increasing use of trawls has reinforcing effects downstream in the value chain since the lower quality meat together with the higher number of smaller crabs landed has sparked a new trend for miniplants in the region. Instead of selling price differentiated product categories, such as “claw,” or “jumbo,” miniplants now sell all the meat together at the same price, making it easier for undersized crab to enter the supply chain. High demand and shortage of catch also incentivizes miniplants to accept smaller crabs and crabs from trawls.

## Discussion

This paper examines the role of strategic agency and leadership by key individuals for the development of a long-running FIP and explores how local social-ecological dynamics interact with the institutional arrangements created by the FIP to affect observed outcomes. Institutional entrepreneurship provides an analytical frame for understanding important processes within FIPs and the conditions under which actions by individuals take shape and affect institutions. In this section, we first discuss the main enabling conditions behind the start of the FIP, highlighting the importance of internal incentive structures, social status and contacts, as well as ecological memory and awareness. We then shed light on the change implementation process, with a focus on how a clear vision for the role and responsibility of industry actors was key for FIP establishment. Finally, we discuss some important barriers for institutional entrepreneurship and FIP strategies, highlighting why these have been unsuccessful in improving the ecological sustainability of fishers’ and traders’ behavior.

### Enabling conditions behind FIP establishment

We found three key enabling conditions that can explain the initiation of the FIP: internal incentive structures; social status and contacts; as well as ecological memory and awareness. First, FIPs are built upon voluntary participation; hence, the incentives for actors to start and be part of FIPs are critical to their success. Market access is often invoked as the main driver and incentive structure behind FIPs (Conservation Alliance 2019; Sampson et al. 2015), and several case studies have described the importance of this as a starting condition (Thomas Travaille, Lindley, et al. 2019; Deighan and Jenkins 2015). However, as seen in other examples of private governance for sustainability, market actors are motivated in part by non-market related factors such as the social license to operate, reduced reputational risk, political power, or moral responsibility (Van Putten et al. 2020; Ponte 2012). For example, Deighan and Jenkins (2015) found that, in their study of Gulf of Mexico reef fish fishery, increased influence in management processes was a key motivation for industry FIP participants to join and engage in the project. Our findings show that rather than market benefits, personal motivation and perceived responsibility together with the decline of crab populations and the perception of a looming ecological crisis were the main triggers for the Indonesian BSC FIP. This supports evidence that market access is not the sole or even the most important driver.

Second, a key individual, and persons around him, was instrumental for the start of the whole crab fishery in Indonesia and for the work towards sustainability within the industry. The central and personal relations that the key individual had with people within the value chain allowed him to emerge as a change agent, by leveraging his social capital (Crona et al. 2017; Bodin and Crona 2008). Although our example of institutional entrepreneurship processes is situated within a market setting, it shows the importance of the social status and contacts as enabling conditions (Battilana et al. 2009). It points to the important ability of socially connected individuals — with the capacity to draw on social relations (i.e., social capital) — to mobilize resources and action (see e.g., Crona et al. 2017; Marín et al. 2012; Aldrich 2012). It also indicates the necessary legitimacy of such individuals to leverage change (Maguire, Hardy, and Lawrence 2004; Crona et al. 2017). While our results show the importance of a key, highly motivated, individual in initiating action, the change processes were ultimately achieved in collaboration with multiple actors at different levels. Enabling processes within the social setting, such as the diversity of organizational forms, and a perceived crisis, allowed for the creation of venues or platforms where key people were able to come together and work towards a common vision through the FIP. From these processes emerged formal institutions, such as industry associations, that then created a credible structure for the work and eventually became change agents themselves. This made it possible for different organizational types to collaborate, such as NGOs, universities, funders, and governmental agencies (Dorado 2005; Westley et al. 2013).

Third, our analysis shows the importance of considering not just the social but also the ecological context in which change is sought, to understand the development and outcomes of the change process in focus. Ecological degradation, together with the awareness and ability to interpret and make sense of the effects of that change (i.e., ecological literacy), has been shown to be important in the emergence of transformation in ecosystem management (Biggs et al. 2010). Our findings show that an understanding of ecosystem dynamics and the ecological memory of fish stock collapse were crucial to trigger an initial response and form an enabling condition for the FIP to emerge. Three ecosystem signals, perceived by actors at different scales, were identified as triggers for behavioral change among value chain actors in the crab fishery and contributed to the establishment and development of the FIP and its outcomes (Fig. 3). The first signal came from the decline in blue crabs in the USA, which led industry leaders to seek other sources of crab. The second was at the national Indonesian level where swimming crab decline triggered the initiation of the FIP. At the level of the fishing village, crab decline led to change in fishing and trading practices. In addition, the ecological characteristics of crabs (e.g., being a fast-growing species), and with the higher market value of larger crabs, both seem to have played a role for incentivizing people to engage with the FIP.

### Actions for change implementation — from developing a vision to changing behavior

By having a clear vision for the role and responsibility of industry actors, the main change agent in the BSC FIP story was able to communicate his personal motivation and conviction to others. We found that his ability to frame the problem, articulate a clear vison for re-organizing the value chain, and create industry associations seems to have resonated with people’s values and willingness, thus substantially contributing to the emergence of the FIP. This creation of a vision to mobilize and motivate others resonates with the strategies highlighted by institutional entrepreneurship scholars as being important for successful change implementation (Battilana et al. 2009). United by this vision, importers and exporters could work towards common sustainability goals and concrete strategies, using the FIP as a platform. The successful implementation of a FIP funding mechanism by the USPA members is another clear example of how the joint vision could mobilize stakeholders to take actions and sustain it over time.

Moreover, the FIP managed to find valuable common ground between conservation and economic interests, which has been discussed as a central contribution for businesses to engage in environmental incentives (Carroll and Shabana 2010). This was because the conservation actions promoted by the FIP were complementary with the processors’ business interests. The alignment between sustainability and business outcomes meant that industry actors had little to lose by supporting the FIP. The vision the FIP had for regulatory change was also likely helped by the overall increased prioritization and attention to sustainable fisheries management in Indonesia, with regulations put in place for several fisheries (CEA 2018), thus paving the way for the management measures promoted by the FIP. It is likely that the growing sustainable seafood movement in North America and EU, with a strengthened demand for sustainable seafood (Roheim et al. 2018), also impacted how companies valued their membership in the industry associations both in the USA and Indonesia, and it may have helped to sustain these new institutions. The continuation of the FIP’s work during the recent COVID-19 crisis is nonetheless a good stress test of the institutionalization of the industry associations and how well the FIP model has worked to create collaborations and improvement at certain levels of the systems (APRI 2021).

However, our examination of the social-ecological dynamics at multiple levels shows that the FIP’s problem framing does not always translate seamlessly to traders and fishers upstream in the value chain. The different ecological and economic changes that local fishers and traders grappled with were not equally accounted for in the formulation of the BSC FIP’s vision, hampering the ultimate sustainability outcomes of the FIP. Combining the institutional entrepreneurship theory with an analysis of complex causality at the local scale adds an empirical understanding of the complexity and struggle that is part of most institutional change processes, which has not been well studied (Hardy and Maguire 2018). Our expansion of the institutional entrepreneurship framework to capture contextual differences that affect different actors’ ability to realize or resist change is therefore essential to understand why seemingly well-resourced sustainability initiatives, with strong institutional buy-in, do not deliver lasting improvements. We elaborate more on these insights below.

### Understanding barriers to institutional entrepreneurship and FIP strategies

Despite the success in aligning business incentives and sustainability-oriented strategies downstream in the value chain, and even contributing to development of regulatory change, over time, the FIP has struggled to curb the trend of declining crab stocks. This is a notable problem for an institutional change process initiated to address the sustainability of the crab fishery. The FIP has navigated this in several ways. They have implemented sourcing policies, worked towards regulations, and regional and local co-management functions. The latest large-scale institutional innovation was the control document. The causal loop diagram shows a range of social and ecological dynamics that impact the ability of the control document to prevent the use of trawls as well as undersized and berried crabs to enter the value chain. These insights begin to shed light on the important and under-analyzed cross-scales aspects of FIP development and performance.

First, our findings highlight some important challenges that global supply chain strategies for sustainable fisheries need to address. Lead firms enjoy significant power because of their control over value chain coordination, which makes many smaller producers and suppliers dependent on them (Gereffi, Humphery, and Sturgeon 2005). In theory, this should increase the leverage of lead firms to change supply chain actors’ behavior (Van Holt and Weisman 2016), but despite this power, our examination of BSC illustrates the difficulties these institutional innovations face in positively impacting social-ecological systems at the local level. Our results emphasize the complex tasks of leveraging change through long value chains spanning geographic and sociocultural divides, even when industry actors are highly motivated. Supply chain strategies that rely on lead firms “governing from a distance” (Boström et al. 2015) may need to find ways to acknowledge and deal with contextual differences among all parts of the supply chain, in order to be effective (Thorlakson et al. 2018).

Second, the findings supports scholarship showing that it is important to consider a combination of economic, social, and environmental factors affecting fishers behavior when attempting to design sustainability initiatives that rely on changing fishers’ behavior (Boonstra et al. 2017; Jentoft 2000, Hauck et al. 2014). It would therefore be important to increase fishers’ and traders’ participation in FIPs, as that can improve the legitimacy of proposed strategies and thus lead to higher compliance and impact overall in projects (Coffey 2005). In addition, proposed ideas to collect social assessment by managers and as part of FIPs (Boonstra et al. 2017; Crona et al. 2019) could be used in combination with initiatives such as control documents. Doddema et al. (2018) argue that data on fisheries behavior need to go beyond conventional monitoring approaches and should include how monitoring interventions interact with the everyday social practices of fishers, not only on vessels, but also on shore. For example, our results show that many fishers in the village preferred to use gear that they could operate single-handed. The FIP could take this perspective from the fishers into account and engage in gear development.

Finally, the case study shows that problems outside the crab value chain itself can have a significant impact on the behavior of actors. These problems can be difficult to target with supply chain strategies. In the BSC case, trawl fishers external to the crab fishery were a main concern for fishers in Betahwalang, but this dynamic was not addressed by the FIP, and not considered within strategies such as control documents. Therefore, FIPs may also need to engage with a wider set of actions, for example, coastal and marine spatial planning processes that address activities outside of the fishery itself, but which can impact the FIP’s success.

## Conclusion

Our in-depth examination of the historical and current development of the Indonesian crab fishery and FIP sheds light on opportunities and barriers for industry leadership towards sustainability in fisheries. It contributes to the scholarly debate around the role of industry actors and voluntary private incentives in environmental governance, as well as to the growing literature around FIPs. We expand the theory of institutional entrepreneurship with three dimensions that are important for understanding shifts towards sustainability. In keeping with existing theory, we illustrate the importance of social position and status as essential for the emergence of institutional entrepreneurship. In addition, ecological memory and awareness were also contributing factors to the establishment of the FIP. We find that industry actors could channel their ambitions to work with environmental issues through the FIP model and how it successfully changed business strategies and national regulations. However, by doing an analysis across the value chain, we discuss why some FIP strategies employed failed to change fishers’ and traders’ behavior. The findings highlight some key challenges for global supply chain strategies for sustainable fisheries. We argue that FIP strategies need to better reflect local contexts and understanding of non-compliance. This could be addressed by more thorough engagement with fishers and traders as key stakeholders in FIP strategy development.

## Supplementary Information

Below is the link to the electronic supplementary material.

Supplementary file1 (DOCX 35.7 KB)

Supplementary file2 (DOCX 14.9 KB)

## Data Availability

Interview data cannot be shared due to confidentiality agreed with interviewees when they gave informed consent to participate.

## Footnotes

1 An exception of the ban for trawls for the northern Java coast region where Betahwalang is located was put in place 2018 because of political pressure (CEA 2018). Although, the government still discouraged the use of trawls through outreach campaigns.

2 In practice, verification of compliance along the value chain means that when fishers land their crab, the first buyer (collector) records the landings and reports the proportion (in kg) of total crab landings that are (1) berried females, (2) under 10 cm, and (3) caught by trawls. Collectors (and other buyers) should then reject crabs in those three categories. The “landing report” should follow the crab catch throughout the whole value chain, and independent audits should be applied along the chain (for more information about the control document, see SFP 2016 and Madduppa et al. 2016).
