# Supplementary material for: From good intentions to unexpected results — a cross-scale analysis of a fishery improvement project within the Indonesian blue swimming crab
Source: Marit Stud. 2022 Oct 6;21(4):587–607. doi: 10.1007/s40152-022-00285-y (PMC9540048; doi:10.1007/s40152-022-00285-y)
Supplement: Supplementary file 1 — Supplementary file1 (DOCX 35.7 KB) [file 40152_2022_285_MOESM1_ESM.docx]

**Appendix 1.** Summary of FIP activities, outputs and workplans for the Indonesian Blue Swimming Crab Fishery Improvement Project

Table 1 shows reported activities on each FIP step until the end of 2019 for the Indonesian Blue Swimming Crab (BSC) FIP. The FIP stages represent the FIP process as a stepwise development with five different stages: 1. FIP development, 2. FIP launch, 3. FIP implementation, 4. Improvements in fishing practices or fishery management, 5. Improvements on the water (Conservation Alliance 2019). Table 2, 3 and 4 summarize FIP workplans from 2013-2022.

**Table 3.** Selection of reported activities for each FIP steps until the end of 2019 (SFP 2022; APRI 2019). For a full list of reported activities see <https://www.fishsource.org/stock_page/742> and APRI 2019.

| **FIP stages** | **Main reported activity** |
| --- | --- |
| 1. **FIP development**   *Fishery assessment or scoping document made public* | July 2009: MSC pre-assessment made public (draft version)   June 2015: MSC Pre-Assessment of Indonesia Blue Swimming Crab |
| 1. **FIP launch**   *Confirmation of project participants and workplan made public* | *June 2009:* FIP participants organized  *July 2012*: Workplan available for 2011 - 2013  *November 2013*: Workplan available for February - December 2013  *January 2014*: Workplan available for 2014 - 2015  *September 2018:* Workplan available for 2016 - 2022 |
| 1. **FIP implementation**   *FIP activities undertaken* | *February 2009:* International Environmental NGO (IENGO) and a company leader met the Director-General of Capture Fisheries to present current work in Indonesia.  *January 2010:* U.S. Crab Processing Association (USPA) sent a letter to the Ministry of Marine Affairs and Fisheries to share their concern for the health of the BSC resource.  *April 2011:* A letter was sent by the Director-General of Fishery Products and Marketing to all Head of Provincial Fishery Offices to inform and encourage the USPA minimum size of crabs as a sourcing policy.  *June 2011:* IENGO and Indonesian Crab Processing Association (IPA) facilitated the workshop “Cost-Effective Stock Assessment for Data Poor Fisheries.”  *July 2011*: USPA minimum size of crabs (8 cm) as a sourcing policy becomes effective *March 2012:* FIP participants meet with the Ministry of Marine Affairs and Fisheries to discuss a management plan.  *November 2012*: USPA adopted a policy that will restrict the purchasing of female crabs bearing eggs.  *February* 2013: IPA and Research Centre for Fish Management and Natural Fishery Conservation (P4KSI) had a Stock Assessment  Workshop.  *September 2013*: Blue swimming crab eggs hatchery has been established in Betahwalang, Demak, and supported by the community.  *March 2014:* Technical meeting with stakeholders to review data collection for calculating Spawning Potential Ratio to implement harvest control in Kendari, together with Indonesia Marine and Climate Support (IMACS) Project (USAID).  *September 2015*: FIP participants meeting to discuss the BSC fishery management plan  *April 2016:* Trial programs for the Control Document  *September 2016:* IPA engages with the government to support BSC sustainability and to establish the Control Document.  *November 2016:* Training on the Control Document and training of independent auditors  *December 2016*: Meeting to discuss the establishment of a BSC Management Committee in East Java, aiming to represent all stakeholders.  *May 2017*: IPA invited by Jepara Fisheries Department on event training of trap making, goal is to distribute traps to small-scale fishers.  June 2017: Focus Group Discussion on certification and quality standards in BSC miniplants.  *July 2018:* Full implement the Control Document system  *October 2018: S*takeholder meetings (fishers, miniplants, government, IPA, IENGO) to develop an understanding of the BSC regulations in Central Java  *October 2018:* IPA shared information about Control Document and traceability systems in seafood product  February 2019: Seed re-stocking completed in South Sulawesi.  May 2019: FIP workshops on BSC harvest strategy  July 2019: Meeting on the socialization of the management plan at the province level |
| 1. **Improvements in fishing practices or fishery management** | *May 2019:* New Village Regulation of PerDes No.02/2019 about BSC management in Betahwalang village was signed. BSC habitat-protected areas were declared by Betahwalang Village (Central Java).  *March 2018:* Increase in MSC Principle Indicator scores ETP species outcome (2.3.1); Habitat management (2.4.2) Habitat information (2.4.3).  *November 2017:* Governor Decree on Establishment of BSC Fishery Management Committee 2017-2022 for the region (Governor of Central Java Decree No. 523/93 2017).  *July 2017:* The Governor Regulation on Fishery Management for Blue Swimming Crab, Lobster and Mud Crab for Central Java Province.  *February 2017*: The Control Document has been independently audited by a third party to assess the implementation - Control Document trials are having a positive effect on adhering to 10cm and berried female standard.  *December 2016:* 2016 Stock Assessment for BSC published  *December 2016:* Government of Indonesia issues management plan for Indonesia Blue Swimming crab K MKP No. 70/KEPMEN-KP/2016.  January 2015: Ministry of Marine Affairs and Fisheries established the Ministerial Decree No. 01/2015 regarding the Minimum Legal Size for Blue Swimming Crab (10 cm)  January 2015: Ministry of Marine Affairs and Fisheries established the Ministerial Decree No. 02/2015 regarding the ban on minitrawl  December 2013: Nursery and Habitat Conservation launched in Demak Regency  September 2013: Fishing gears exchanged from trawl to collapsible trap: 2800 traps have been handover to fishers. |
| 1. **Improvements on the water** | December 2016: Increase in MSC BMT (Benchmarking and  Tracking Tool) scores on Secondary species outcome (2.2.1) |

**Summary of workplans

Summary of workplan actions 2016-2022**The full workplan on fisheryprogress.org is divided into the 28 indicators following the MSC standard. Here we present the main activities divided into the three overall MSC principles (Indonesian BSC 2016-2022). For full workplan see [https://fisheryprogress.org/node/5081/improvement#](https://fisheryprogress.org/node/5081/improvement) .

**Table 4**. Summary of FIP workplan activities 2016-2022 (APRI 2018).

| MSC Principle 1: Sustainability of the fish stock. *“There are enough fish left in the sea to reproduce so fishing can continue indefinitely” (MSC 2022)* | | |
| --- | --- | --- |
| **Main activity** | **Tasks** | **Responsible Parties** |
| Stock Assessment Portunus pelagicus in Indonesia | a. Hire and train enumerators; b. Data collection and analysis; c. Online database; d. Scientist meeting | Indonesian Crab Processing Association (IPA), Research Centre for Fish Management and Natural Fishery Conservation (P4KSI), Ministry of Marine Affairs and Fisheries (MMAF) |
| Stock Enhancement dan Restocking (Portunus pelagicus) | Spread seed of Blue Swimming Crab at several locations | IPA, BBPBAP Jepara, Balai Besar Perikanan Budidaya Air Payau(Brackish Water Aquaculture Center) |
| Contribute to the development of harvest strategy for Indonesia BSC | Conduct workshop on Harvest Strategy and Harvest Control Rule | IPA, P4KSI, MMAF |
| Fishing Effort Studies | a. Data collection of fishing gear b. Online database c. Scientist meeting | IPA, P4KSI, MMAF |
| Contribute to the development of harvest strategy for Indonesia BSC | Conduct workshop on Harvest Strategy and Harvest Control Rule | IPA, P4KSI, MMAF |
| MSC Principle 2: minimizing environmental impacts *“Fishing operations must be carefully managed to maintain the structure, productivity, function, and diversity of the marine ecosystem” ( MSC 2022)* | | |
| **Main activity** | **Tasks** | **Responsible Parties** |
| Non-Target Species Field Assesment - Using the MSC's Risk Based Framework for Data Limited Fisheries | a. Hire and train enumerators for doing ecological impact survey and enumeration  b. Data collection and logbook  c. Data analysis  d. Fishing gear exchange  e. Data collection for crab habitat distribution  f. Data collection for fishing ground  g. Seagrass and coral reefs monitoring  h. Establish habitat protection | IPA, P4KSI, MMAF |
| Habitat and Ecosystem Impact: Genetic Portunus pelagicus | a. Sample collection  b. Genetic analysis  c. Data collection for crab habitat distribution  d. Seagrass and coral reefs monitoring  e. Establish habitat protection | IPA, USPA |
| MSC Principle 3: effective management  *“The fishery must comply with relevant laws and have a management system that allows it to respond quickly to changes in the status quo” (MSC 2022)* | | |
| Establish Indonesia BSC fisheries Co-Management at the local level | a. Conduct meetings at national provincial, district and village levels b. Develop and implement agreed action plans | Indonesian Crab Processing Association (IPA), U.S. Crab Processing Association (USPA), International Environmental NGO (IENGO), MMAF,  Province and village districts of the government |
| Implement control document to improve compliance and traceability | a. Miniplant mapping  b. Trial control document  c. Control document implementation to improve compliance and traceability and etc. | IPA, USPA, IENGO, MMAF, Province and village districts of government |

**Summary workplan 2014-2015**

**Table 5.** Summary of FIP workplan 2014-2015 (APRI 2015)**.**

| **Topic** | **Activity** | **Responsible actor** |
| --- | --- | --- |
| Stock  Assessment  (Java Sea) | Lit review | Research Centre for Fish Management and Natural Fishery Conservation (P4KSI) |
|  | Assessment proposal | P4KSI |
|  | Enumerator training | Indonesian Crab Processing Association (IPA) , P4KSI |
|  | Data collection | IPA, P4KSI |
| Stock Assessment (Kendari) | Data collection | IPA |
|  | Complete SPR (Spawning Potential Ratios) assessment | Indonesia Marine and Climate Support (IMACS) Project (USAID) |
| Stock Management (Kendari) | Harvest Control Rules; Target reference point; Harvest Strategy | Data Management Committee (DMC) Sulawesi |
| Fishery management (Java Sea) | Annual stakeholder review | IPA, MMAF |
|  | Management framework | MMAF |
| Fishery management (Kendari) | Data Management Committee (DMC) meeting to review data and management inputs | IPA |
| Consultancies | MSC Gap Review | IPA, MRAG |
|  | MSC pre-assessment Kendari | IMACS Project (USAID) |

**Summary workplan 2013**

**Table 6.** Summary of FIP workplan activities 2013 (SFP 2013).

| **Topic** | **Activity** |
| --- | --- |
| Stock assessment in the major fishing ground defined | Design stock assessment method, identify sampling areas and improve data collection with Research Centre for Fish Management and Natural Fishery Conservation (P4KSI) |
|  | Existing enumerators collect data weekly for a full year |
|  | Get support from Indonesia Marine and Climate Support (IMACS) Project in SE Sulawesi for stock assessment through the Spawning Potential Ratio (SPR) approach |
|  | Workshop to review Indonesia BCS stock status |
| Management | Continue to help the government to draft BSC Fishery Management Plan |
| Crab Biology and Ecological Study | Research on crab biology and ecology. Work with BBPPI  (Fishing Technology Development Centre) |
|  | Present research findings to local and national government authorities |
| Enhancement | Study the effectiveness of stock enhancement program including development of protocols of crab releasing and monitoring |
| Holding Berried Females | Study and monitor the effectiveness of holding berried females |
| Education | Develop a community-based pilot project to include community fisheries management and education in Demak (Central Java) with a potential partner of Diponegoro University |
|  | Results and analysis of community-based fisheries management pilot project should be used for developing BSC management plan document |

**References**

APRI. 2015. “APRI FIP Work Plan 2014-2015.” https://www.apri.or.id/full-width-page-layout/downloads/download-info/apri-fip-work-plan-2014-2015/. A

———. 2018. “Indonesia BSC FIP Workplan-2016-2022.” https://fisheryprogress.org/node/5081/info.

———. 2019. “Fishery Improvment Program 2008-2019.” https://www.apri.or.id/full-width-page-layout/downloads/download-info/apri-fip-2008-2019/.

Conservation Alliance ( The Conservation Allience for Seafoof Solution. 2019. “Guidelines for Supporting Fishery Improvement Projects.” http://solutionsforseafood.org/wp-content/uploads/2019/09/FIP_report_screen-final_revised_september.pdf

SFP. 2013. “Indonesia Blue Swimming Crab Workplan 2013.”

———. 2022. “Blue Swimming Crab Java Sea. FishSource Profile. In: FishSource [Online]. Updated 31 October.” 2022. https://www.fishsource.org/stock_page/742.
