# Supplementary material for: From good intentions to unexpected results — a cross-scale analysis of a fishery improvement project within the Indonesian blue swimming crab
Source: Marit Stud. 2022 Oct 6;21(4):587–607. doi: 10.1007/s40152-022-00285-y (PMC9540048; doi:10.1007/s40152-022-00285-y)
Supplement: Supplementary file 2 — Supplementary file2 (DOCX 14.9 KB) [file 40152_2022_285_MOESM2_ESM.docx]

| **Method of data collection** | **Actor type or type of activity** | **Number** | **Follow up interviews** |
| --- | --- | --- | --- |
| Semi-structured interviews | Importer | 4 | 1 |
|  | Processing association representative | 5 | 4 |
|  | NGO representative | 4 | 4 |
|  | Enumerator | 1 | 2 |
|  | Industry trader | 1 | 3 |
|  | Local processor | 2 |  |
|  | Collector | 5 |  |
|  | Fisher | 22 | 2 |
| Focus group | Fishers and collectors | 1 (4 traders and 4 fishers) |  |
| Participatory observations | Fishing boat trip, Indonesia | 4 |  |
|  | Landing site and market, Indonesia | 6 |  |
|  | Fisher group meeting, Indonesia | 2 |  |
|  | Local processing site, Indonesia | 1 |  |
|  | Seafood trade show, USA | 1 |  |
|  | Maryland Crab restaurant, USA | 1 |  |
|  |  |  |  |

**Appendix 2.** Method of data collection

**Table 7.** Method of data collection and actor type or type of activity involved.
